# The Risks of Inappropriateness in Cardiac Imaging

**DOI:** 10.3390/ijerph6051649

**Published:** 2009-05-14

**Authors:** Eugenio Picano

**Affiliations:** Institute of Clinical Physiology, CNR, Pisa, Italy; E-Mail: picano@ifc.cnr.it

**Keywords:** appropriateness, benefit, radiation, risk

## Abstract

The immense clinical and scientific benefits of cardiovascular imaging are well-established, but are also true that 30 to 50% of all examinations are partially or totally inappropriate. Marketing messages, high patient demand and defensive medicine, lead to the vicious circle of the so-called *Ulysses syndrome*. Mr. Ulysses, a typical middle-aged “worried-well” asymptomatic subject with an A-type coronary personality, a heavy (opium) smoker, leading a stressful life, would be advised to have a cardiological check-up after 10 years of war. After a long journey across imaging laboratories, he will have stress echo, myocardial perfusion scintigraphy, PET-CT, 64-slice CT, and adenosine-MRI performed, with a cumulative cost of >100 times a simple exercise-electrocardiography test and a cumulative radiation dose of >4,000 chest x-rays, with a cancer risk of 1 in 100. Ulysses is tired of useless examinations, exorbitant costs. unaffordable even by the richest society, and unacceptable risks.

## 1. Introduction

**Figure 1.**
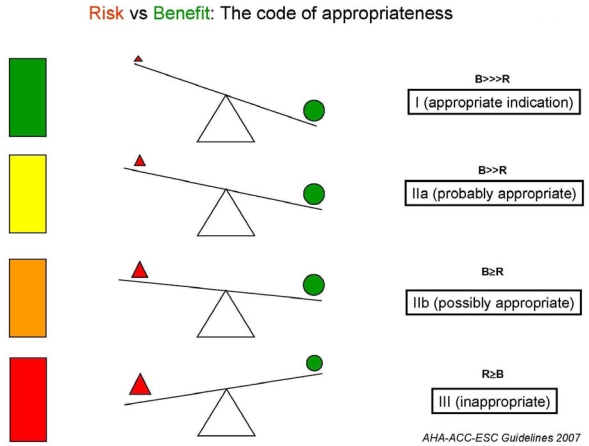
The balance between risks (red triangle) and benefits (green circles) determining the appropriateness score of testing. The three corners of the red triangle represent acute, subacute, and long-term (radiation) risks. Acute risks occur within seconds and minutes (for instance, death or myocardial infarction during stress or cath); sub-acute risks within days or weeks (for instance, contrast-induced nephropathy); and long-term risks (due to cumulative exposure to ionizing radiation) after years or decades.

Every year five billion imaging tests are performed worldwide, and about half of these are cardiovascular examinations [1]. According to recent estimates, at least one-third of all examinations are partially or totally inappropriate, i.e. risks and costs outweigh benefits [2]. A survey of the Italian Association of Echocardiography showed that 50% of resting transthoracic echocardiograms are totally or partially inappropriate [3]. The inappropriateness rate of coronary angiography has been described to be as high as 65% in some tertiary care referral centers [4]. The inappropriate testing rate in high-volume, tertiary care referral center was 28% for stress echo [5] and 36% for stress perfusion scientigraphy [6], and rose to 43% when no screening of external referral was implemented [5]. In a Finnish CT study, up to 77% of CT examinations of the lumbar spine were not justified [7]. The number of appropriate cardiac CT examinations is still today around 20%, and was around 30% a few years ago [8]. Following the definition of the American College of Cardiology Foundation, an appropriate imaging study is one in which the expected incremental information, combined with clinical judgement, exceeds any expected negative consequences by a sufficiently wide margin for a specific indication that the procedure is generally considered acceptable care and a reasonable approach for the indication [9] (Figure 1). Negative consequences include the risks of the procedure itself (i.e., radiation or contrast exposure) and the downstream impact of poor performance such as delay in diagnosis (false negatives) or inappropriate diagnosis (false positives). This implies potential harm for patients undergoing imaging (who take the risks of an imaging study without a commensurate benefit), excessive delay in the waiting lists for other patients needing the examination and an exorbitant cost for society, with no improvement and possibly with a reduction in care quality [9].

As recently pointed out by Redberg, health care costs in the United States now exceed two trillion dollars, representing 16% of the country’s gross domestic product by 2016, and, in the words of Greenspan, are on “an unsustainable trajectory” [10]. Since 1993 cardiac imaging in individuals covered by Medicare has increased at an annual rate of 6.1%. Diagnostic imaging has increased more rapidly than any other component of medical care [4]. The volume of cardiovascular services increased 5.5% per capita between 2004 and 2005 in the United States, driven largely by the growth of cardiac imaging services [10]. Although the diagnostic and prognostic information provided by these tests is not without a cost, some studies have shown that the use of non-invasive imaging in appropriately selected patients translates into savings because of more appropriate selection of even more expensive procedures [11,12]. However, these studies involved patients who were appropriately selected for testing; and the trade-off between costs and benefits will not be the same when studies are performed less appropriately [13]. In order to limit the detrimental consequences of the pandemic of inappropriateness and diagnostic obesity, the U.K. College of Radiology in 1999 [14], the European Commission in 2001 [15], and more recently the American College of Cardiology [9] have prepared guidelines on appropriateness of general or specialized imaging testing, including stress perfusion scintigraphy [16] and stress echocardiography [17]. The ultimate goal of these documents is to define the appropriate test for the appropriate indication in the appropriate patient: a difficult, elusive and moving target which is however one of the new features, and not the least important, of good quality medical care [9,15].

## 2. The Ulysses Syndrome in the Cardiac Imaging Lab

**Figure 2.**
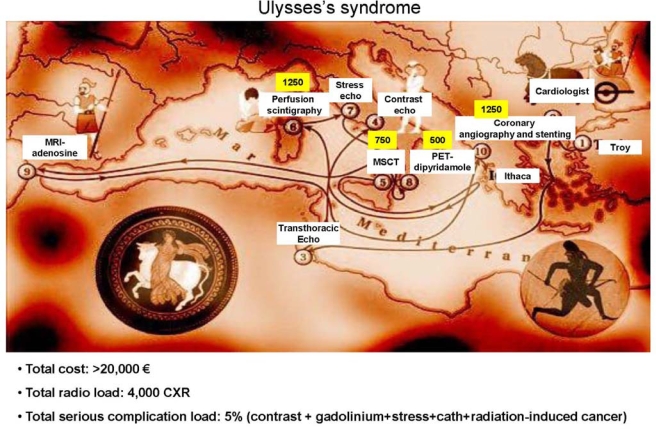
Ulysses’ voyage as a metaphor for the diagnostic pathway of the patient with suspected coronary artery disease. At the end of the first round of this odyssey, the cumulative cost is more than 100 times a simple exercise-electrocardiography. The cumulative radiation dose is that of more than 4,000 chest x-rays. The cumulative damage (including acute, subacute, and long-term risks) will cause a serious health detriment (including infarction, renal insufficiency, or cancer) in about 5–10% of patients.

The Ulysses syndrome was first described in 1972 by Canadian physician Dr. Mercer Rang, who applied it to the ill effects of extensive diagnostic investigations conducted because of a false-positive or indeterminate result in the course of a routine laboratory screening [18]. Ulysses left Troy in full physical and psychological health. Equipped with a safe ship and a competent crew, he was sure he would return home quickly; instead it turned out that he lost all his crew, his ship and he was able to make it home only after a journey full of hardships. Today, the most frequent diagnostic investigation is a cardiac imaging test. Mr. Ulysses, a typical middle-aged “worried-well” asymptomatic subject with an A-type coronary personality, a heavy (opium) smoker, leading a stressful life, would be advised to have a cardiological check-up after 10 years of war (Figure 2). In an asymptomatic patient, routine screening even with a simple exercise testing is not indicated (class III). The indication is IIB (weak supportive evidence) for asymptomatic men older than 45 years with multiple risk factors and/or who are involved in occupations in which impairment might impact public safety, according to 2003 recommendations of the American College of Cardiology/American Heart Association [19]. The family physician directly refers the patient to the cardiologist (step 2) who suggests a transthoracic echocardiogram (step 3), which is perfectly normal, but with poor visualization of segment 17, the true apex. The patient is again sent to the echo lab to repeat the transthoracic echo with echo-contrast injection (step 4): the apex is perfectly visualized and looks normal. However, just to be on the safe side, the cardiologist suggests a multislice computed tomography (step 5). Ulysses accepts enthusiastically since he recently read the front page and cover story of Time Magazine (September 5, 2005) explaining that in this way you can detect asymptomatic life-threatening coronary artery stenosis. The scan shows only minor luminal irregularities of very uncertain pathological meaning. At this point, thallium stress perfusion scintigraphy (step 6) is performed. A very mild, questionable hypoperfusion of the infero-basal wall is documented. The stress echo (step 7) is performed and a very mild apical hypokinesis is observed at peak exercise in presence of marked systolic blood pressure rise. At this point the cardiologist asks for further examinations and Mr. Ulysses is becoming increasingly anxious. One after another, Ulysses undergoes a PET-adenosine stress (step 8: marginally positive at basal lateral wall) and MRI adenosine with gadolinium contrast (step 9: marginally positive on the basal inferior septum). The patient is eventually referred to coronary angiography (step 10); the island of Ithaca is crowded with non-significant coronary stenoses, unrelated to perfusion defects or wall motion abnormalities, which may, however, trigger the oculo-stenotic reflex [10] leading to the vicious circle of angioplasty (obviously with drug-eluting stent), imaging test for the diagnosis of silent re-stenosis, presence of perfusion or wall motion defects, re-angiography, and so on and so forth.

None of these examinations are free, and each implies a financial and a safety cost. For a resting cardiac imaging test, taking the average cost (not charges) of an echocardiogram as equal to 1 (as a cost comparator), the cost of a CT is 3.1x, of a SPECT 3.2x, of a cardiovascular magnetic resonance imaging 5.51x, of a PET scan 14.03x and of a right and left heart catheterization 19.95x [20]. For stress cardiac imaging, compared with the treadmill exercise test considered as equal to 1 (as a cost comparator), the cost of a stress scintigraphy is 2.1x, and of a stress SPECT scintigraphy 5.7x [19].

There are non-negligible acute risks in several non-invasive imaging techniques. Exercise testing entails a very small but definite risk, and data confirm that up to 1 myocardial infarction or death per 2,500 tests occurs [19]. Major, life-threatening side effects (sustained ventricular tachycardia, ventricular fibrillation and myocardial infarction) occur in about 1 out of 300 dobutamine-echocardiography and 1 out of 1,000 dipyridamole-echocardiography tests [21–23]. In general, exercise is safer than pharmacological stress, in which major complications are three times more frequent with dobutamine than with dipyridamole [24,25].

The use of nephrotoxic contrast in large doses with radiologic imaging is a major concern, since it induces an acute worsening of renal function – not always reversible – in about 10% of patients with impaired renal function [26]. Also with echocardiography, the use of contrast may add an extra risk of about 1 in 10,000 of allergic and life-threatening [27] reactions, and – experimentally – the number of injured cardiomyopathies increased with increasing contrast agent dose and ultrasound exposure [28], possibly due to acoustic cavitation and acoustic current production which may locally deliver high levels of mechanical energy (higher with increasing power output, mirrored by the mechanical index). Minor side effects (probably due to microembolization of injected bubbles) are frequent in up to 10% of patients, and include headache, nausea and vomiting, dysgeusia and dyspnea. Major life-threatening side effects are rare but possible, and consist of ventricular tachycardia, pulmonary edema, ventricular fibrillation and even death. Toxic effects led the FDA to force manufacturers to add a “boxed warning” on Luminity in October 2007, stating that “4 deaths and 110 various non-fatal reactions occurred during or 30’ after the infusion (out of about 2 million studies)” [28,29]. Recently, additional concern arose regarding the use of paramagnetic contrast during CMR in patients with impairment of renal function. Paramagnetic contrast agents have long been considered absolutely safe and well tolerated. However, in May 2007, the FDA (and EMEA) ordered a boxed warning on the safety of gadolinium-containing contrast agents, on the basis of 200 cases of nephrogenic systemic fibrosis (NSF) that occurred in patients with kidney failure. NSF (also called nephrogenic fibrosing dermopathy, NFD) causes the patient to develop tight and rigid skin making it difficult to bend joints. NSF/NFD may also result in fibrosis, or sclerosis, of body organs resulting in the inability of body organs to work properly and can lead to death [30]. Risks are acute (linked to stress), subacute (linked to contrast use) and long-term (linked to radiation): Table 1.

The rate of complications is obviously higher with invasive imaging procedures. For instance, coronary angiography has a cumulative risk of 1–2% of major complications (including dissection, myocardial infarction, stroke) and 1 in 1,000 risk of death [26]. Contrast-induced nephropathy is the third most common cause of hospital-acquired renal failure, ranging between 3 and 14% in patients with cardiovascular pathology undergoing angiography procedures [26]. All of these risks may be fully acceptable in presence of a proper indication, but become totally unacceptable if the indication is less than appropriate. More than 10 million stress imaging procedures [31] and more than one million coronary angiographies [31] are performed every year in the USA alone. The small individual risk thus becomes an important population burden.

**Figure 3.**
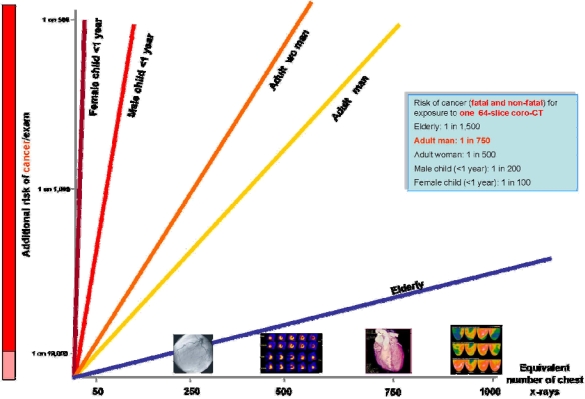
On the x axis, the doses of 4 common imaging examination are shown: coronary angiography (250 chest x-rays); myocardial perfusion scintigraphy (500 chest x-rays); 64-slice CT (750 chest x-rays); whole body CT- PET scan (1,250 chest x-rays). On y-axis risk for children (male and female), adults (men and women) and the elderly. Redrawn and modified from ref. [35].

Besides these clearly recognized acute and subacute risks, long-term risks linked to imaging radiation should also be considered [32]. Medical x-rays and γ-rays are a proven human carcinogen [14,15,33]. In radiology and nuclear medicine, higher acute doses correspond to higher long-term risks; there are no safe doses, and all doses add up in determining the cumulative risks over a lifetime [14,15,34]. Doses of common imaging are reported in yellow in Figure 2, and range from the equivalent of 300 chest x-rays of a coronary angiography to that of 1,250 chest x-rays of a thallium or PET-CT scan [35]. With imaging cumulative doses (radiation expenditure), the patient “buys” increasing risks of developing cancer during their lifetime. The estimated risk of developing cancer, based on the Seventh Report of the authorative Committee to Assess Health Risks from Exposure to Low Levels of Ionizing Radiation (BEIR VII report) linear nothreshold model [33], is presented in Figure 3.

As an example, the attributable risk of cancer for a multislice computed tomography give a dose equivalent to about 750 chest x-rays with a lifetime extra-risk of 1 cancer in 750 of exposed adult males, 1 in 500 of exposed adult females, and 1 in 100 in female children aged less than 1 year.

In other words, at the end of the first round of examinations shown in Figure 2, Ulysses has paid about 100 times the cost of a simple exercise-electrocardiography test – probably even that not needed [19]. He received a 5% cumulative risk of major short-term adverse events (from renal insufficiency to myocardial infarction). He received a cumulative dose exposure of about 4,000 chest x-rays, corresponding to an extra-risk of cancer of 1 in 150. The invasive and interventional procedures that he received did not improve his quality of life since he was asymptomatic at the beginning of his cardiological history and the anatomy-driven revascularization will not increase his life expectancy [19,36,37]. Periodic follow-up examinations with imaging testing will be scheduled – mostly inappropriately [15,6] - and the Odyssey will last probably forever.

## 3. Appropriateness in Cardiac Stress Imaging

**Figure 5.**
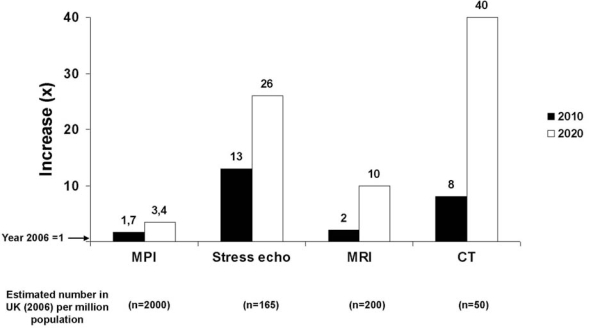
Future trends in the use of cardiac imaging up to the year 2020. Redrawn from the original data of reference [35].

The proliferation of cardiac stress imaging may represent an added value when appropriate, and an added cost when inappropriate. Unfortunately, the definition of appropriateness is obvious in theory, but not so straightforward on practical grounds. Unlike prevention and treatment strategies supported by evidence-based practice guidelines, the evidence base for imaging is anecdotal, fragmented, and lacking in prospective clinical trials [9]. As a consequence, the process for developing appropriateness criteria is only partially evidence-based and is heavily weighted by expert consensus [9]. On an arbitrary scale of 1 (most inappropriate) to 9 (most appropriate), indications are classified as “appropriate” (score >7, test is generally acceptable and is a reasonable approach for the indication), “uncertain” (score between 4 and 6, test may be generally acceptable and may be a reasonable approach for the indication), and “inappropriate” (score <3, test is not generally acceptable and is not a reasonable approach for the indication). The most frequent appropriate, uncertain and inappropriate indications met in the clinical practice of high volume laboratories are listed in Table 2. Following these criteria, only 2 out of 3 stress echo (or nuclear stress imaging) tests are appropriate, with similar numbers observed in disparate geographic, cultural and economic situations – from Italy to Australia [5] to the USA [6]. Of interest, the vast majority of inappropriate studies were restricted to only a few patient indications, with the four most frequent inappropriate indications listed in Table 2 account for 88% of all inappropriate examinations [38]. This repetitive pattern of inappropriateness points to a need for quality improvement and educational programs to achieve measurable improvement in results [38]. This is especially important today and in view of the projected spectacular rise of cardiac imaging in the next 15 years [39] (Figure 5).

It is certainly good to have multiple imaging tools, which allow us to avoid the contraindications and limitations of each technique and to tailor the best (most effective) test in individual patients. The best test choice should also consider the test with the lowest cost – for any given accuracy – and, importantly, the test with the lowest acute, chronic and long-term risks. This concept is clearly spelled out in guidelines of the U.K. College of Radiology in 1999 [24], Medical Imaging Guidelines in 2001 [15], and the American College of Cardiology Guidelines in 2006 [16,17].

For instance, with special regard to the radiation issue, European Union guidelines state that “for instance, because MRI does not use ionizing radiation, MRI should be preferred when both CT and MRI would provide similar information and when both are available”. The American College of Cardiology definition of appropriateness defines an imaging study as “one in which the expected incremental information exceeds the negative consequences, which include the risks of the procedure (i.e., radiation or contrast exposure) and the downstream impact of poor test performance”.

Imaging technology evolution in the last 30 years was characterized by a shift from 1-dimensional (1970s) to a 2-dimensional (1980s–90s) and current 3-dimensional representation of the heart provided by all major imaging modalities. Probably, the evolution of technology has not always been fully matched by our maturity in using it. We should probably move from a 1-dimensional approach to imaging (typical of the 1980s) to the 2-dimensional approach (considering cost-effectiveness, not only effectiveness) up to a three-dimensional approach integrating benefit, cost, and risk.

Appropriateness in health care, like quality, can be a moving target and not easy to define. However, it is also true that, as with many quality measures, the very act of having appropriateness criteria and measuring your own appropriateness performance is likely to improve the quality of what is being measured [38]. This needs to be done to improve the quality of our profession, to address the existing concerns of those who pay for these services, and to optimize the immense benefits our patients can derive from the appropriate practice of cardiac imaging [40].

## 4. The Risks of Unawareness

Good medical practice warrants knowledge of the doses and long-term risks of these tests – which can be judiciously employed when they are most appropriate. Unfortunately, recent evidence shows that such radiological awareness is uncommon. The results of a survey performed on British physicians shows that one out of 20 doctors does not realise that ultrasound does not use ionizing radiation, that one out of 10 does not realise that magnetic resonance imaging does not use ionizing radiation, and 97% of doctors grossly underestimate (on average by sixteen-fold) the doses of radiation for most commonly requested investigations [41]. Another survey on Israeli orthopedists shows that the mortality risk of radiation induced carcinoma from bone scan was identified correctly by less than 5% of respondents and senior orthopedists estimated lower risks than did residents [42]. A third survey was performed on Emergency Room physicians and radiologists in a U.S. academic centre. Almost all doctors were unable to accurately estimate the dose for a computed tomography scan compared with that for one chest radiograph. Among radiologists, 5% of respondents thought that a computed tomography scan dose was less than one chest radiograph, and 56% estimated the computed tomography scan dose between 1 and 10 chest radiographs, with dramatic underestimation of the true dose (about 500 chest radiographs) [43]. In another survey conducted in a tertiary care referral centre of adult and pediatric cardiology, Correia *et al*. found that 95% of physicians wrongly underestimated the risk of fatal cancer associated with a stress myocardial perfusion scintigraphy. The correct dose of a stress sestamibi myocardial scintigraphy (corresponding to 500 chest x-rays) was correctly estimated by 29% of physicians, whereas 71% wrongly estimated the dose exposure as equal to one (13% of respondents), or one half (9%) or three times (49%) that of a chest x-ray [44]. If risks are ignored, all examinations become appropriate: benefit without risk. The dose information should be expressed clearly for each and every examination, especially in cardiology, where many examinations with high radiation dose are performed many times. Table 3 summarizes doses and risks of common cardiological examinations. Doses are reported from European Union Medical Imaging guidelines [15] and Royal College of Radiology 2007 [45], and coronary stenting and thallium scan doses updated from latest American Heart Association document [40]. This information is something that everyone, including patients, should know, and almost everyone – including physicans - ignore.

## 5. The Risks of Technology Underuse

Advances in imaging technology have led to an explosive growth in the performance of cardiovascular imaging. This growth is challenging since it may lead overuse or inappropriate use of new technology. However, underuse of this technology is equally inappropriate. Radiation protection should not cause examinations needed to be refused or not performed. Justification is probably best made by a physician who is familiar with the patient’s medical history [46], and both justification and dose optimization are best implemented if a regular audit program is a part of the clinical practice [47]. However, the regular application of audit, awareness and appropriateness implementation is usually not facilitated by a health system that pays for volumes, not for appropriateness. New payment models should be developed to pay physicians more for providing clearly appropriate procedures and substantially less for procedures of limited value. Although it is certain that this is more easily said than done, there is no doubt that a system paying the quality, not only the quantity, of the procedures would be an enormous boost to appropriateness.

## 6. Conclusions

The benefits of diagnostic imaging are immense and have revolutionized the practice of medicine. The use of radiological investigations is an accepted part of the medical practice justified in terms of clear clinical benefits to the patient, and failure of providing services from which the patient would likely benefit is dangerous. However, the unjustified use of diagnostic testing can be equally dangerous.

## Figures and Tables

**Figure 4.**
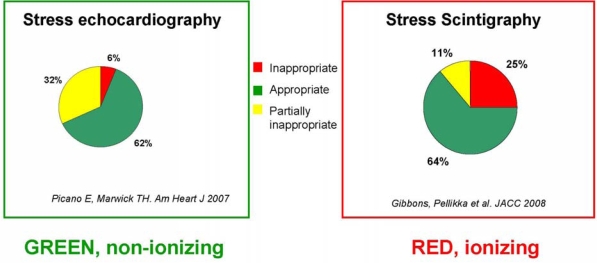
Inappropriateness in stress echocardiography (*left*) and cardiac stress imaging (*right*). Data are derived from [5] (Pisa and Brisbane echo labs in Italy and Australia) and [6] (Mayo Clinic nuclear cardiology lab in the USA).

**Table 1.** Acute, subacute, and long-term risks in cardiac imaging.

	**ACUTE**	**SUBACUTE**	**CHRONIC**

**Most frequent cause**	Stress	Iodinated contrast	Radiation
**Timing**	Seconds	Days	Years
**Examples**	Myocardial infarction	Renal failure	Cancer
**Cellular target**	Endothelium of coronary arteries	Kidney tubular cell	Somatic cells (lung, breast, bone marrow)
**Risk per exam**	1 in 500-1 in 1,000	1 in 50-1 in 100	1 in 500-1 in 1,000
**Cumulative nature**	No	No	yes

**Table 2.** Most frequent appropriate/uncertain/inappropriate indications of cardiac stress imaging (with echo or nuclear) in CAD detection and/or risk stratification.

	**Appropriate**	**Uncertain**	**Inappropriate**
ECG uninterpretable or unable to exercise, or prior stress ECG equivocal	√		
Coronary artery stenosis of unclear significance (CT or angio)	√		
Post-revascularization not in the early post-procedure period, with change in symptoms	√		
Pre-surgery, high risk non-emergent, poor exercise tolerance <4 METS	√		
Viability (dobutamine) Ischemic cardiomyopathy, known CAD, pt eligible for revascularization	√		
Asymptomatic or stable symptoms, repeat stress echo after > 5 yrs		√	
Asymptomatic < 5 yrs post CABG or < 2 yrs post-PCI		√	
Asymptomatic, low risk			√
Pre-op, intermediate risk surgery, good exercise capacity			√
Symptomatic, low pre-test probability, interpretable ECG, able to exercise			√

Pre-op, low-risk surgery

**Table 3.** Dose/Risk Communication The Royal College of Radiologists approach (RCR)

**Investigation**	**Effective Dose (mSv)**	**Equivalent No. of Plain Chest Radiographs**	**Approximate Equivalent Period of Natural Background Radiation**	Additional Lifetime Risk of Fatal and Non-Fatal Cancer*	RCR Symbolic Representation**
Plain PA chest radiograph	0.02	1	3 days	1:1,000,000	
Lung perfusion scintigraphy (Tc99m)	1	50	6 months	1:10,000	
CT chest (non contrast)	8	400	3.6 years	1: 1,200	
Perfusion cardiac Rest-stress Technetium 99m sestamibi scan	10	500	4 years	1:1,000	
MDCT Cardiac (64-slice)	15	750	7 years	1:750	
Coronary stenting	20	1050	8 years	1:500	
Thallium-201 scan	41	2000	16 years	1:250	

* These examples relate to a 50 year-old male. Multiply by 1.38 for women, by 4 for children under 1 year, and by 0.5 in an 80 year old male;

** : <1 mSv, : 1 – 5 mSv, : 5 – 10 mSv, : > 10 mSv; On the right side column, symbology proposed by Royal College of Radiology, 2007 [45].
